# Protein phosphorylation regulation of key genes in oral squamous cell carcinoma and their role in the immune microenvironment

**DOI:** 10.3389/fimmu.2025.1677807

**Published:** 2025-09-26

**Authors:** Yu Zeng, Chunyang Wang, Wenli Zhao, Ye Zhao

**Affiliations:** ^1^ Department of Stomatology, Tianjin Medical University General Hospital, Tianjin, China; ^2^ Department of Scientific Research, Tianjin Medical University General Hospital, Tianjin, China; ^3^ Institute of Chinese Medicine, Yanting County People’s Hospital, Yanting, China; ^4^ International College, Krirk University, Bangkok, Thailand

**Keywords:** oral squamous cell carcinoma, FN1, multi-omics analysis, immune microenvironment, molecular subtyping

## Abstract

**Background:**

Oral squamous cell carcinoma (OSCC) is one of the most common malignant tumors in the head and neck region, with a complex molecular mechanism that has not yet been fully elucidated. This study aims to identify key genes closely associated with the development and progression of OSCC through integrative multi-omics data analysis and to explore the potential roles of these genes in protein phosphorylation regulation and the immune microenvironment, providing new insights for precision diagnosis and treatment.

**Methods:**

The study integrated data from National Center for Biotechnology Information (NCBI) and National Institutes of Health (NIH) sources, combining differential expression gene analysis and co-expression network construction to identify candidate genes significantly associated with phosphorylation status. Key genes were further screened, and molecular subtyping of samples was performed based on gene expression patterns. Additionally, the association between key genes and immune microenvironment characteristics was evaluated, and Mendelian randomization (MR) was employed to investigate the impact of genetic variants on disease risk.

**Results:**

The analysis revealed multiple significantly differentially expressed genes, primarily enriched in pathways related to cell cycle regulation, signal transduction, and metabolism. Five key genes—BMP2, FN1, INHBA, MMP9, and THY1—were ultimately identified. These genes exhibited subtype-specific expression patterns across different molecular subtypes and were closely associated with immune cell infiltration levels. Furthermore, functional validation demonstrated that FN1 was significantly linked to OSCC occurrence at the genetic level.

**Conclusion:**

This study identified key genes and molecular subtypes associated with OSCC, highlighting their potential links to protein phosphorylation and the immune microenvironment. Among these, FN1 may serve as a potential risk gene and a candidate biomarker, providing novel insights into the molecular mechanisms of OSCC.

## 1 Introduction

Oral Squamous Cell Carcinoma (OSCC) is the most common malignant tumor in the head and neck region and ranks sixth in terms of incidence among malignant tumors worldwide (1). It is estimated that in 2020, approximately 400,000 people worldwide were affected by oral epithelial cancer, resulting in about 178,000 deaths. This disease ranks 16th among global cancer incidence and mortality rates (2, 3). Among oral specialty diseases, OSCC accounts for over 90% of all oral malignant tumors. The 5-year survival rate of Chinese patients is only 50-60% (4, 5). In addition, the prevalence of OSCC varies significantly around the world: it is the most common type of cancer in Southeast Asian countries, while it ranks 16th in Finland (6). This global disparity in prevalence rates is mainly attributed to the varying degrees of exposure among populations to carcinogenic risk factors such as tobacco, including smoking and smokeless tobacco products (7). In addition, alcohol exposure, betel nut chewing habits, HPV infection and gene mutations are all important pathogenic factors of OSCC (8). OSCC is a multi-stage disease that usually progresses from the initial normal mucosa to a potential malignant disease in the oral cavity, and eventually becomes invasive carcinoma (9). In the early stage, the patient presents with persistent oral ulcers, white and red spot lesions and local pain. In the late stage, it is often accompanied by lymph node metastasis in the neck. OSCC is often diagnosed only when the disease has progressed to an advanced stage, which results in a lower five-year survival rate for affected patients (10). Although surgery remains the main treatment for OSCC, there is still controversy over the surgical management of cervical lymph nodes in patients, especially on whether to intervene in the contralateral neck (11). Although targeted drugs and immune checkpoint inhibitors have been applied in clinical practice, their overall response rates remain relatively low (12, 13). This therapeutic predicament mainly stems from the high heterogeneity of tumors, the suppression of the immune microenvironment and the drug resistance mechanism (14). Therefore, in-depth analysis of the molecular regulatory mechanism of OSCC, especially the epigenetic modification network such as protein phosphorylation, has an urgent need to improve the prognosis of patients.

Protein phosphorylation is a key post-translational modification of proteins, which regulates the function of proteins by adding a phosphate group to a specific amino acid on the protein molecule, such as serine, threonine or tyrosine (15). This process plays a crucial role in cellular signal transduction as it can alter the activity, localization, stability of proteins and their interactions with other molecules (16). In cancer biology, dysregulation of protein phosphorylation is regarded as one of the significant factors leading to tumorigenesis and development (17, 18). Many studies have shown that in various types of cancer, the activities of protein kinases and phosphatases in certain signaling pathways have undergone significant changes (19). For instance, the abnormal activation of classic oncogenic signaling pathways such as EGFR/RAS/RAF/MEK/ERK and PI3K/AKT/mTOR is usually closely related to changes in protein phosphorylation levels (20). The overactivity of these pathways can promote cell proliferation, inhibit apoptosis, enhance invasiveness and metastasis ability, and contribute to the development of the tumor microenvironment, thereby advancing the cancer process (21, 22).

In this study, by integrating multi-source gene expression data, the key phosphorylation molecular pathways and candidate genes closely related to the occurrence and development of OSCC were systematically identified. By integrating functional enrichment analysis with molecular feature mining, we identified multiple core factors with potential regulatory roles in tumor progression and revealed their biological significance in the immune microenvironment. Based on the heterogeneity analysis of gene expression patterns, we classified the patients into subgroups with different molecular characteristics, suggesting their potential value in disease classification and individualized treatment. The research results provide a theoretical basis for in-depth exploration of the phosphorylation molecular mechanism of OSCC and the development of new intervention targets.

## 2 Materials and methods

### 2.1 Data acquisition and preprocessing

The bulk RNA sequencing data of OSCC and their corresponding clinical data — GSE9844 (12 Normal tissue vs 26 Tumor tissue), GSE30784 (45 Normal tissue vs 167 Tumor tissue), GSE74530 (6 Normal tissue vs 6 Tumor tissue), GSE78060 (4 Normal tissue vs 26 Tumor tissue), and GSE138206 (18 Tumor tissue) are from the National Center for Biotechnology Information (NCBI). Additionally, we have downloaded OSCC data from the National Institutes of Health (NIH). Records of patients with missing information were excluded. The sequencing data was converted into Transcripts Per Million (TPM) format for subsequent analysis. If the data distribution is highly scattered, a log2 transformation of the expression matrix was performed.

This study focuses on the analysis of phosphorylation-related genes in OSCC. The relevant gene sets are from: GOBP_CARBOHYDRATE_PHOSPHORYLATION.v2025.1.Hs.gmt, GOBP_DEPHOSPHORYLATION.v2025.1.Hs.gmt, GOBP_LIPID_PHOSPHORYLATION.v2025.1.Hs.gmt, GOBP_OXIDATIVE_PHOSPHORYLATION.v2025.1.Hs.gmt, GOBP_PHOSPHOLIPID_DEPHOSPHORYLATION.v2025.1.Hs.gmt, GOBP_PHOSPHORYLATION.v2025.1.Hs.gmt, GOBP_REGULATION_OF_PHOSPHORYLATION.v2025.1.Hs.gmt. We take the intersection of these gene lists and include the intersected genes for further analysis.

### 2.2 Differential gene expression analysis and weighted gene co-expression network analysis analysis

Differential gene expression (DEG) analysis was performed on NIH and NCBI datasets using the criteria of P < 0.05 and |log2FoldChange| > 1. Results were visualized with volcano plots, highlighting significantly up- and down-regulated genes.

For WGCNA, phosphorylation-related scores were first calculated for each sample based on the intersected phosphorylation-related gene set. A weighted gene co-expression network was then constructed using the WGCNA R package. The optimal soft-threshold power (β = 5) was selected based on scale-free topology criteria and mean connectivity, ensuring the resulting network approximated a scale-free topology while maintaining sufficient connectivity among genes. Gene modules were identified using hierarchical clustering and the dynamic tree cut algorithm, with minimum module size set to 30 genes. Modules showing significant correlation with phosphorylation-related scores (Pearson correlation, |R| > 0.2, P < 0.05) were selected for subsequent analyses, consistent with standard WGCNA procedures (23). Module-trait relationships were visualized using a module-feature heatmap.

### 2.3 Intersection genes and their biological function analysis

We use the “Venn” package to create Venn diagrams, taking the intersection of the differential genes from the NCBI data, the differential genes from the NIH data, the genes from the WGCNA-selected modules, and the phosphorylation-related genes. Based on the intersected genes, we conduct biological function analysis, including Gene Ontology (GO) analysis and Kyoto Encyclopedia of Genes and Genomes (KEGG) enrichment analysis using the “clusterProfiler” package, and visualize the results with bar charts. Additionally, we use wilcox.test to calculate the expression differences of the intersected genes between the tumor and normal groups in both the NIH and NCBI databases, and display the differential analysis results of the intersected genes using box plots from the “ggpubr” package.

### 2.4 Comprehensive analysis of key gene selection using machine learning methods

To ensure the reliability of the results, we applied three machine learning methods—Least Absolute Shrinkage and Selection Operator (LASSO) regression, Support Vector Machine (SVM), and Random Forest—to comprehensively screen candidate genes for key gene identification. The following R packages were used during the analysis: “randomForest,” “xgboost,” and “caret.” In the LASSO analysis, the optimal regularization parameter λ was determined through k-fold cross-validation. To balance the model’s predictive accuracy and complexity, we selected λmin as the regularization parameter for the final model and used it for gene selection. In the SVM, we selected the points with the highest accuracy, smallest error, and fewest features for gene screening. In the Random Forest, we selected genes with an importance greater than 3 for further analysis. For the screening results from the three methods, we used a Venn diagram to take the intersection, identifying several key genes.

### 2.5 SHapley Additive exPlanations-based model interpretation and validation

/For the NIH and NCBI source data, we first randomly divided the data into training and validation sets in a 7:3 ratio. Subsequently, we performed SHAP analysis on the models built using the three machine learning methods to assess the contribution of each feature to the prediction results. The following R packages were used in the analysis: “kernelshap,” “ggplot2,” “ranger,” and “shapviz.” Specifically, based on the SHAP values of the key genes, we assessed the relative importance of the key genes in the model’s prediction results and visualized them using a variable importance heatmap. Additionally, we revealed the interdependencies and potential complex interactions of different key genes in the model prediction using variable dependence plots. We also used variable accumulation attribution waterfall charts to visually display the cumulative contribution of each key gene to the model’s prediction results. Furthermore, we evaluated the model performance under different machine learning methods in both the training and validation sets using four metrics: Area Under the Curve (AUC), Recall, Precision, and F1_Score, and selected the method with the best performance for further analysis.

### 2.6 Molecular subtype analysis based on non-negative matrix factorization clustering

Unsupervised clustering was performed on key gene expression data using the Non-negative Matrix Factorization (NMF) algorithm. The optimal number of clusters was determined based on the cophenetic correlation coefficient and residual sum of squares (RSS), which are widely used metrics to assess NMF cluster stability (24). Two molecular subtypes (C1 and C2) were chosen, balancing cluster stability and biological interpretability.

Differential expression analysis of key genes among the subtypes was conducted using the “limma” R package. Immune infiltration of 28 immune cell types across subtypes was quantified and visualized using boxplots generated with the “ggpubr” package. Gene Set Variation Analysis (GSVA) was applied to examine pathway activation differences between subtypes. Finally, Principal Component Analysis (PCA) was performed to validate the discriminative power of the identified subtypes across datasets.

### 2.7 Immune microenvironment features and correlation analysis of key genes with immune cells

To assess immune cell infiltration, we applied single-sample Gene Set Enrichment Analysis (ssGSEA) to quantify the relative abundance of 28 immune cell types. The corresponding immune cell–specific gene signatures were obtained from the C7 (immunologic signatures) collection of the Molecular Signatures Database (MSigDB, v7.5.1). After completing the overall immune microenvironment assessment, we focused on the key genes identified and explored their potential correlations with various immune cells in the tumor patient population. For the correlation analysis, we primarily used the “limma” and “ggplot2” software packages for statistical analysis and graphical visualization.

### 2.8 Mendelian randomization analysis

To investigate the potential impact of genetic variation on OSCC, we conducted a systematic study using MR analysis. All analyses were performed using the “TwoSampleMR” package in R, with strict settings for linkage disequilibrium (LD) pruning: P-value threshold of 5×10^-8^, r² = 0.001, and an F-statistic greater than 10 to ensure the validity and independence of the selected instrumental variables.

In this study, we used OSCC data from the Finnish Biobank as the outcome variable, and the exposure factors were based on the expression quantitative trait loci (eQTL) of the key genes in the blood. After performing LD pruning, only a few genes retained valid Single Nucleotide Polymorphism (SNP) sites, which were used for subsequent analysis.

Our analysis first used a forest plot to clearly demonstrate the effect size and direction of each SNP site on OSCC outcomes. Then, scatter plots were used to further analyze the relationships between SNPs, exposure factors, and their effects on the outcome variable. Five mainstream MR methods (Inverse variance weighted, MR Egger, Simple mode, Weighted median, and Weighted mode) were used for robustness testing, ensuring the reliability of the analysis results. To further verify the stability of the analysis and detect potential heterogeneity or bias, we performed a leave-one-out sensitivity analysis. Additionally, funnel plots were applied to assess the symmetry of instrumental variables, providing a preliminary judgment of the risk of publication bias. Finally, a comprehensive MR analysis was conducted on the FN1 gene to explore its association with OSCC.

### 2.9 Clinical sample collection and preparation

Tumor and paired normal tissues were collected from fifteen OSCC patients during surgeries at Tianjin Medical University General Hospital between May 2019 and April 2024. Normal tissues were obtained from areas at least 3 cm away from the tumor margins. Following surgical excision, tissue samples were immediately snap-frozen in liquid nitrogen and stored at −80°C to preserve RNA integrity for subsequent molecular analyses. Ethical approval for the study was obtained from the Institutional Review Board (IRB) of Tianjin Medical University General Hospital, and written informed consent was provided by all patients prior to tissue collection.

### 2.10 Cell culture and siRNA transfection

Human oral squamous cell carcinoma (OSCC) cell lines (OKF5, FaDu, SCC-9, SCC-25, HSC-3, and HSC-2) were used in this study. Cells were cultured in DMEM medium supplemented with 10% fetal bovine serum (FBS) at 37°C in a 5% CO_2_ incubator, with regular changes of fresh medium. Subsequently, small interfering RNA (siRNA) was used to transfect OSCC cells to knock down the expression of the FN1 gene. The experimental group was transfected with siRNA specific to FN1 (si-FN1), while the control group was transfected with non-specific control siRNA (si-NC). The transfection was performed according to the instructions of the Lipofectamine 3000 reagent, and subsequent experiments were conducted 48 hours after transfection.

### 2.11 qPCR detection of FN1 mRNA expression

Total RNA was extracted from cells using Trizol reagent and reverse transcribed into cDNA according to the instructions of the reverse transcription kit. Using the cDNA as a template, qPCR reactions were performed with specific primers. The reaction conditions were as follows: initial denaturation at 95°C for 5 minutes, followed by 40 cycles of denaturation at 95°C for 15 seconds and annealing at 60°C for 30 seconds. GAPDH was used as an internal reference, and the relative expression level of FN1 was calculated using the 2^-^▵▵Ct method.

### 2.12 Transwell assay for cell migration and invasion

Under 4°C conditions, Matrigel was diluted in serum-free medium (1:8), and 50-60μL was evenly applied to the upper surface of the bottom membrane of the Transwell chamber, followed by incubation at 37°C for 1–3 hours. After incubation, excess liquid was removed, and 100μL of serum-free medium was added for hydration at 37°C for 30 minutes. Cells were starved for 12–24 hours, resuspended in serum-free medium, and adjusted to a density of 5×10^5^/mL. 100μL of cell suspension was added to the upper chamber, while 600μL of medium containing 10% FBS was added to the lower chamber, and the cells were cultured for 12–48 hours. Subsequently, the cells that had migrated through were detected. Five random fields were selected under the microscope for counting, and ImageJ software was used for quantitative analysis.

### 2.13 Western blot detection of apoptosis- and EMT-Related proteins

Cells were lysed with RIPA lysis buffer to extract total protein, and the protein concentration was determined using the BCA protein concentration determination kit according to the instructions. The protein samples were separated by SDS-PAGE, transferred to PVDF membranes, and the membranes were blocked with 5% skim milk for 1 hour. The membranes were then incubated with primary antibodies (FN1, cleaved caspase-3, Bcl-2, E-cadherin, Vimentin, β-actin) at 4°C overnight, followed by incubation with secondary antibodies for 1 hour at room temperature. The proteins were visualized using ECL chemiluminescence, and the band densities were analyzed using ImageJ software. Additionally, cell apoptosis was detected using the Annexin V-FITC/PI double staining kit. Transfected cells were collected and stained with Annexin V-FITC and PI according to the kit instructions, and the apoptosis rate was determined using flow cytometry.

### 2.14 Statistical analysis

All statistical analyses were conducted using R software (version 4.1.3). In Receiver Operating Characteristic (ROC) analysis, an AUC greater than 0.6 was considered to have good diagnostic efficacy. Correlation analysis, unless otherwise specified, used Pearson correlation methods. A *P* -value less than 0.05 was considered statistically significant (*P<0.05; **P<0.01; ***P<0.001; **** *P*<0.0001).

## 3 Results

### 3.1 DEG analysis and WGCNA analysis

**Figure 1:**
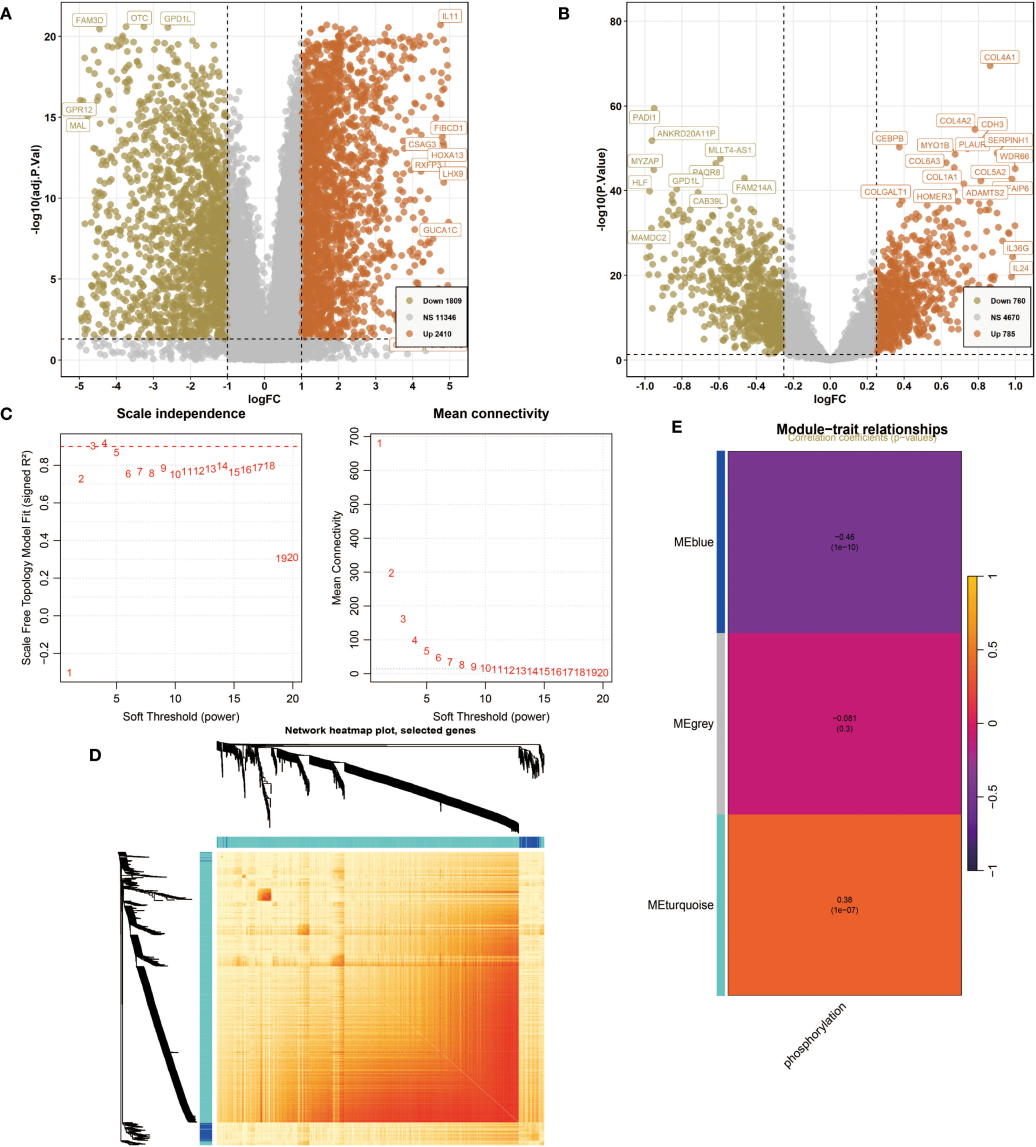
Differentially expressed genes (DEG) analysis and weighted gene co-expression network analysis (WGCNA). **(A)** The volcano plot shows DEGs of Oral squamous cell carcinoma (OSCC) in National Institutes of Health (NIH) data. *P*<0.05 and |log2FoldChange|>1 were identified as significant DEGs. The downregulated genes (Down) = 1809, upregulated genes (Up) = 2410. **(B)** The volcano plot shows DEGs of OSCC in National Center for Biotechnology Information (NCBI) data. *P*<0.05 and |log2FoldChange|>1 were identified as significant DEGs. The downregulated genes (Down) = 760, upregulated genes (Up) = 785. **(C)** Determination of the optimal soft threshold power for constructing a scale-free network. (Left) Scale independence analysis. (Right) Mean connectivity analysis. **(D)** Network heatmap plot of selected genes. **(E)** Module characteristic heatmap showing the correlation between module features and phosphorylation-related scores.

First, for the NIH source data, DEG analysis revealed 1,809 downregulated genes (e.g., FAM3D, OTC, GPD1L, GPR12, MAL) and 2,410 upregulated genes (e.g., IL11, FIBCD1, CSAG3, HOXA13, RXFP3, LHX9, GUCA1C) (**Figure 1A**). For the NCBI source data, the volcano plot showed 760 downregulated genes (e.g., PADI1, ANKRD20A11P, MLLT4-AS1, MYZAP, HLF, GPD1L, PAQR8, FAM214A, CAB39L, MAMDC2) and 785 upregulated genes (e.g., COL4A1, COL4A2, CDH3, CEBPB, MYO1B, PLAUR, SERPINH1, WDR66, COL1A1, COL5A2, COLGALT1, HOMER3, ADAMTS2, IL36G, IL24) (**Figure 1B**). Next, we conducted WGCNA. Based on Scale independence and Mean connectivity, we selected β = 5 as the optimal soft threshold, where R² ≈ 0.85, and the network displayed good scale-free properties and moderate sparsity (**Figure 1C**). Using the WGCNA co-expression network, we successfully identified 3 modules (**Figure 1D**). The module feature heatmap showed that MEblue and MEturquoise were highly correlated with phosphorylation-related scores (**Figure 1E**). Therefore, we included the genes from these two modules in subsequent analysis.

### 3.2 Intersection genes and their biological function analysis

**Figure 2:**
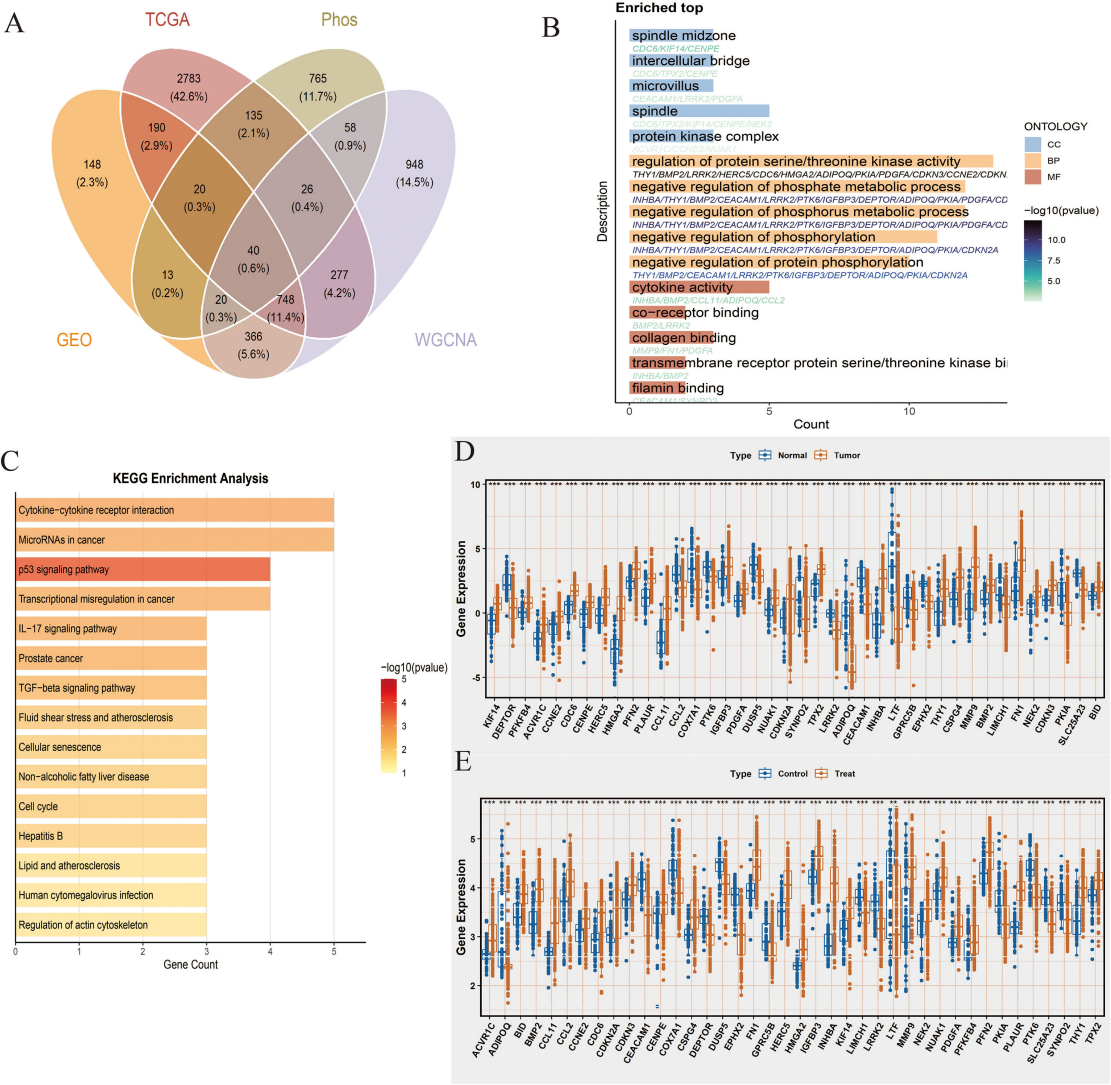
Intersection genes and their biological function analysis. **(A)** Venn diagram showing the intersection of differential genes from NCBI (n=260 tumor vs. 73 normal), NIH (n=241 tumor vs. 57 normal), WGCNA module genes significantly correlated with phosphorylation-related scores (|R|>0.2, P<0.05), and phosphorylation-related genes, resulting in 40 overlapping genes. **(B)** Gene Ontology (GO) enrichment analysis of the 40 intersection genes. The top enriched biological processes, cellular components, and molecular functions are shown. Adjusted P values (Benjamini-Hochberg correction) <0.05 were considered significant. Enrichment scores represent -log10(adjusted P value). **(C)** KEGG pathway enrichment analysis of the 40 intersection genes. Only pathways with adjusted P < 0.05 are displayed; enrichment scores are shown as -log10(adjusted P value). **(D)** Boxplots comparing expression levels of the 40 intersection genes between tumor and normal samples in the NIH dataset (tumor n=241, normal n=57). Statistical significance was assessed using Wilcoxon rank-sum test; *P < 0.05, **P < 0.01, ***P < 0.001, ****P < 0.0001. **(E)** Boxplots comparing expression levels of the 40 intersection genes between tumor and normal samples in the NCBI dataset (tumor n=260, normal n=73). Statistical analysis as in **(D)**.

/Using the “Venn” package, we took the intersection of the differential genes from NCBI, NIH, WGCNA module genes, and phosphorylation-related genes, successfully identifying 40 intersection genes (**Figure 2A**). We then performed enrichment analysis on these 40 intersection genes. GO analysis revealed that they were significantly enriched in pathways related to cell division (Spindle, Spindle midzone, Intercellular bridge), signal transduction (Protein kinase complex, Regulation of protein serine/threonine kinase activity, Negative regulation of phosphorylation, Negative regulation of protein phosphorylation, Transmembrane receptor protein serine/threonine kinase binding), and metabolic regulation (Negative regulation of phosphate metabolic process, Negative regulation of phosphorus metabolic process). KEGG analysis indicated that they were mainly enriched in pathways related to immune and inflammatory responses (Cytokine-cytokine receptor interaction, IL-17 signaling pathway, TGF-beta signaling pathway), cancer progression (MicroRNAs in cancer, p53 signaling pathway, Transcriptional misregulation in cancer, Prostate cancer), and metabolic and endocrine regulation (Non-alcoholic fatty liver disease, Lipid and atherosclerosis, Fluid shear stress and atherosclerosis) (*P*<0.05, **Figures 2B, C**). Finally, box plots demonstrated the gene expression differences of the 40 genes in different databases. These genes showed significant differences between the two groups in both data sources. Specifically, in the NIH source data, the expression level of LTF was significantly higher in the Normal group compared to the Tumor group, whereas genes such as HMGA2 and FN1 were expressed at higher levels in the Tumor group (**Figure 2D**). In the NCBI source data, LTF was also significantly overexpressed in the Control group, while genes such as BMP2 and CDKN2A showed higher expression levels in the Tumor group (*P*<0.001, **Figure 2E**).

### 3.3 Comprehensive analysis of key genes selected by machine learning methods

**Figure 3:**
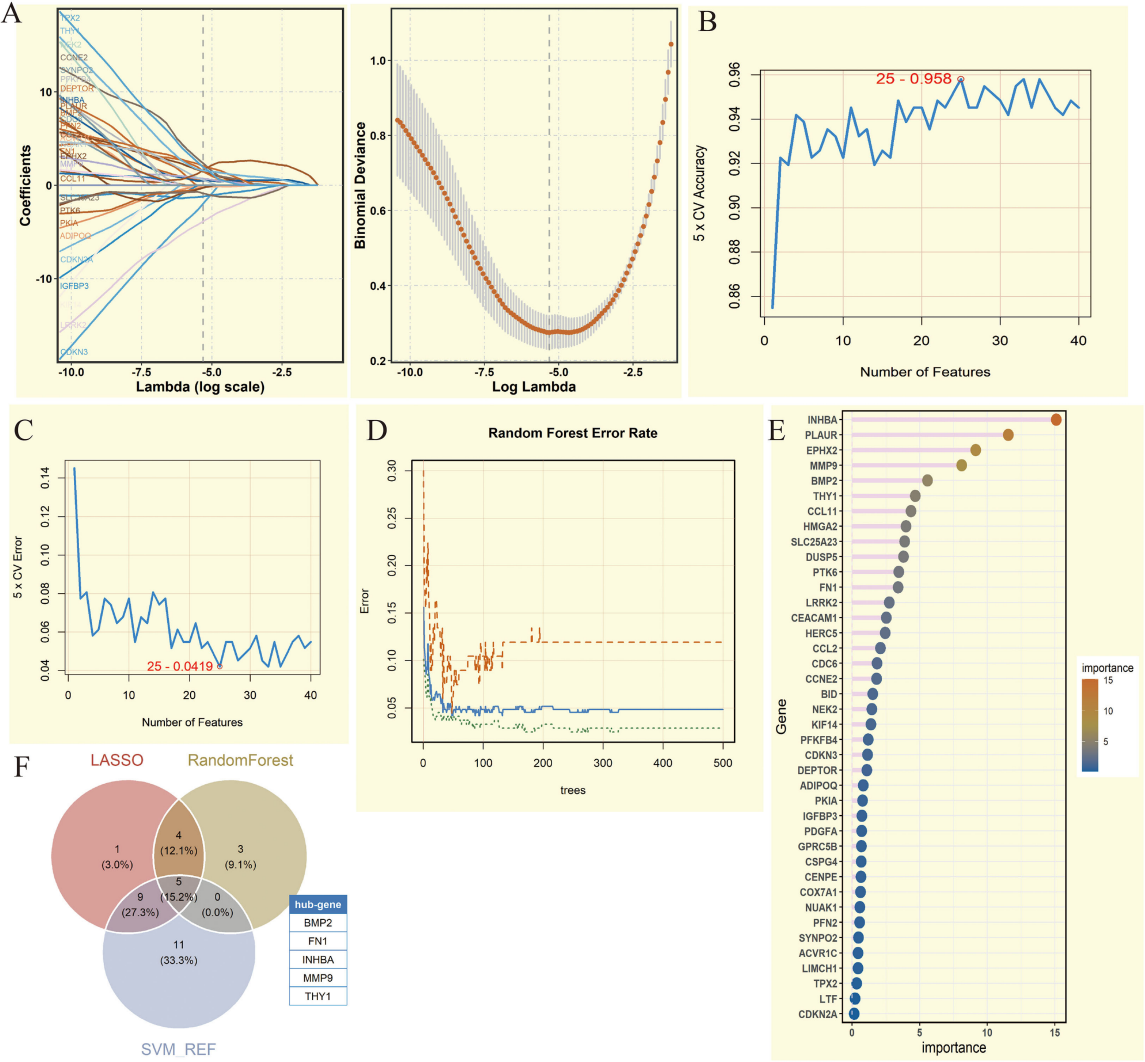
Comprehensive analysis of key genes screened by machine learning methods. **(A)** Coefficient path diagram and deviation curve of Least Absolute Shrinkage and Selection Operator (LASSO) regression analysis. **(B)** 5-fold Cross-Validation (5 x CV) accuracy change of Support Vector Machine (SVM) under different feature numbers. **(C)** 5-fold Cross-Validation (5 x CV) error change of SVM analysis under different feature numbers. **(D)** Error rate change of Random Forest analysis under different numbers of trees. **(E)** Lollipop chart of gene importance in Random Forest analysis. **(F)** Venn diagram showing the intersection of genes from three machine learning methods. Five genes were obtained (BMP2, FN1, INHBA, MMP9, and THY1).

To identify key candidate genes, we applied three complementary machine learning methods: LASSO regression, SVM, and RF. First, LASSO regression, based on the regularization path and 10-fold cross-validation, selected 19 significant genes (**Figure 3A**). Next, SVM was applied, and when the number of features was set to 25, the model achieved its highest accuracy of 0.958 (**Figures 3B, C**), resulting in 25 candidate genes. Finally, Random Forest analysis was performed, and 12 genes with importance scores greater than 3 were retained, with INHBA exhibiting the highest importance score (**Figures 3D, E**). To integrate results from the three methods, we constructed a Venn diagram, which revealed that five genes—BMP2, FN1, INHBA, MMP9, and THY1—were consistently selected across all approaches. These five genes were considered key genes and were used for subsequent analyses, ensuring robustness and reliability in the screening strategy.

### 3.4 SHAP-based model explanation and validation in different datasets

**Figure 4:**
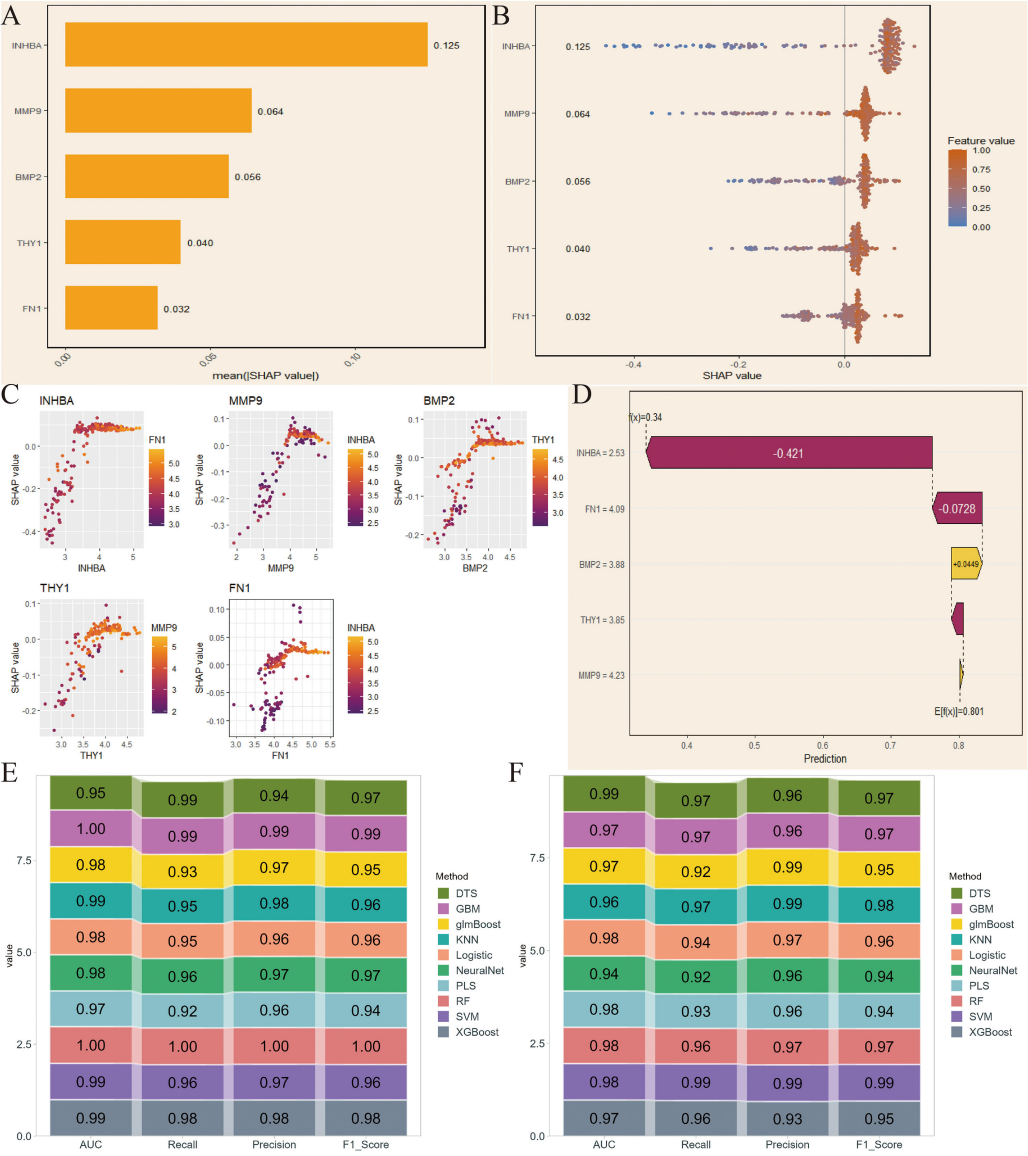
SHapley Additive exPlanations (SHAP)-based model interpretation and validation in NCBI data. **(A)** SHAP values of the 5 key genes. **(B)** Variable importance hexagonal chart of the 5 key genes. **(C)** Variable dependency plot of the 5 key genes. **(D)** Cumulative attribution waterfall plot of the 5 key genes. **(E, F)** Evaluation of the model performance of 10 machine learning algorithms based on AUC, Recall, Precision, and F1_Score.

We first applied SHAP in the NCBI source data to obtain SHAP values for the 5 key genes, with INHBA showing the highest importance and FN1 showing relatively low importance (**Figure 4A**). The variable importance honeycomb plot further validated this conclusion (**Figure 4B**). We then found that as the expression level of a key gene increased, its contribution to the model’s prediction became more stable and significant, especially when multiple key genes were highly expressed at the same time. This revealed the complex interaction mechanism of key genes in model prediction (**Figure 4C**). Furthermore, we visualized the cumulative contribution of the 5 key genes to the model’s prediction results using the variable accumulation attribution waterfall plot. It can be seen that INHBA had the most significant negative contribution, while other genes had relatively smaller contributions, and BMP2 had a positive effect (**Figure 4D**). Additionally, we evaluated the model’s performance under different machine learning methods using AUC, Recall, Precision, and F1_Score. The evaluation results showed that RF performed excellently in these indicators’ ROC results, indicating that it could provide stable and efficient prediction capabilities (**Figures 4E, F**).

**Figure 5:**
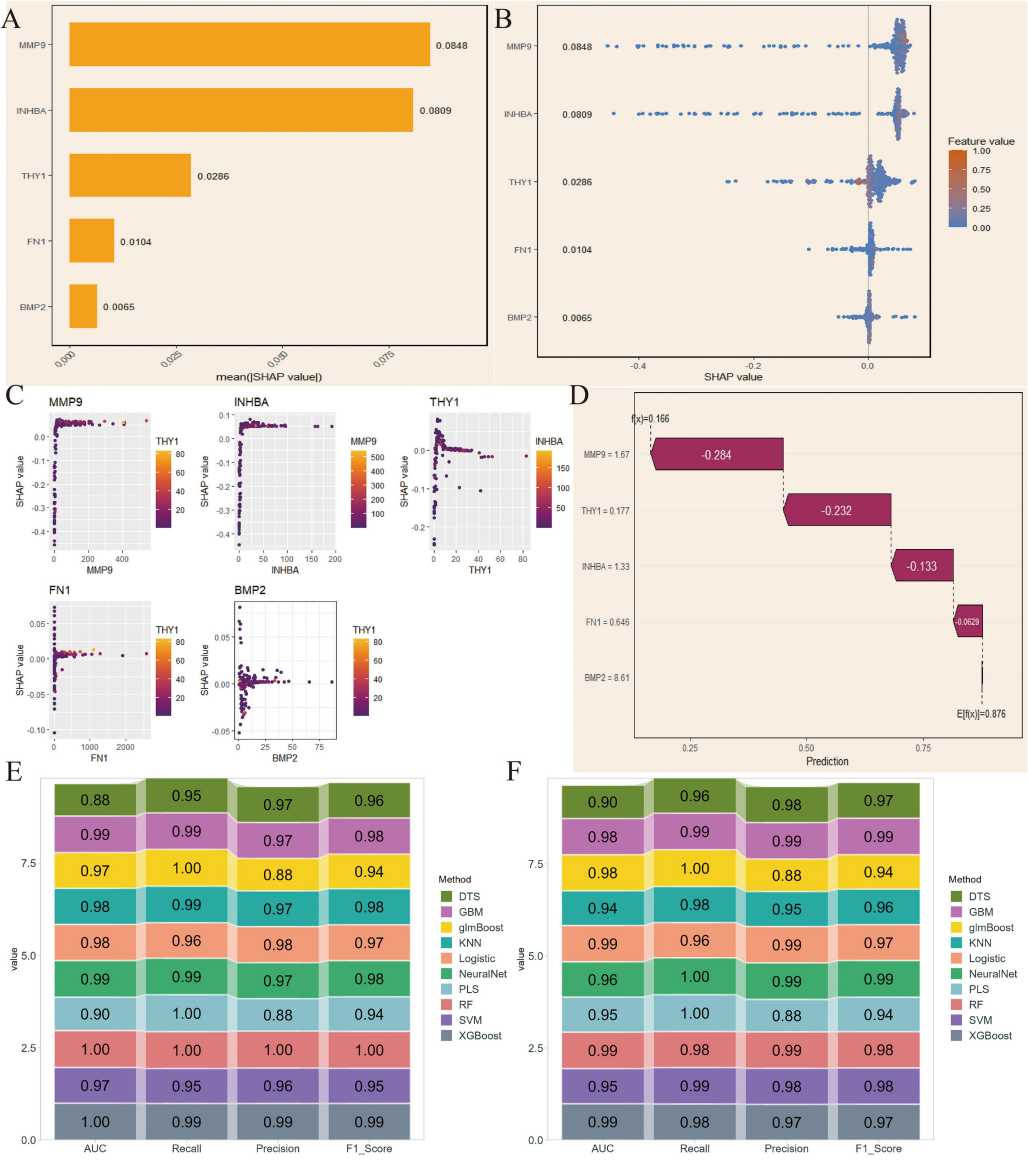
SHAP-based model interpretation and validation in NIH data. **(A)** SHAP values of the 5 key genes. **(B)** Variable importance hexagonal chart of the 5 key genes. **(C)** Variable dependency plot of the 5 key genes. **(D)** Cumulative attribution waterfall plot of the 5 key genes. **(E, F)** Evaluation of the model performance of 10 machine learning algorithms based on AUC, Recall, Precision, and F1_Score.

Next, we conducted the same analysis on the NIH source data to verify the reliability of the selected key genes. By comparing SHAP values and the variable importance honeycomb plot, we found that MMP9 and INHBA had good importance, consistent with the NCBI source data results (**Figures 5A, B**). The variable dependence plot also displayed the relationship and interaction between the expression levels of the core genes and their SHAP values for model prediction contribution. Similarly, the SHAP values of INHBA and THY1 showed a significant correlation with their respective expression levels, and were influenced by other genes such as MMP9 and INHBA (**Figure 5C**). Moreover, except for BMP2, the core genes had a negative contribution to the model’s prediction results (**Figure 5D**). Finally, the ROC results validated that RF had excellent model performance (**Figures 5E, F**). We selected RF for subsequent analysis.

### 3.5 Molecular subtype analysis based on NMF clustering

**Figure 6:**
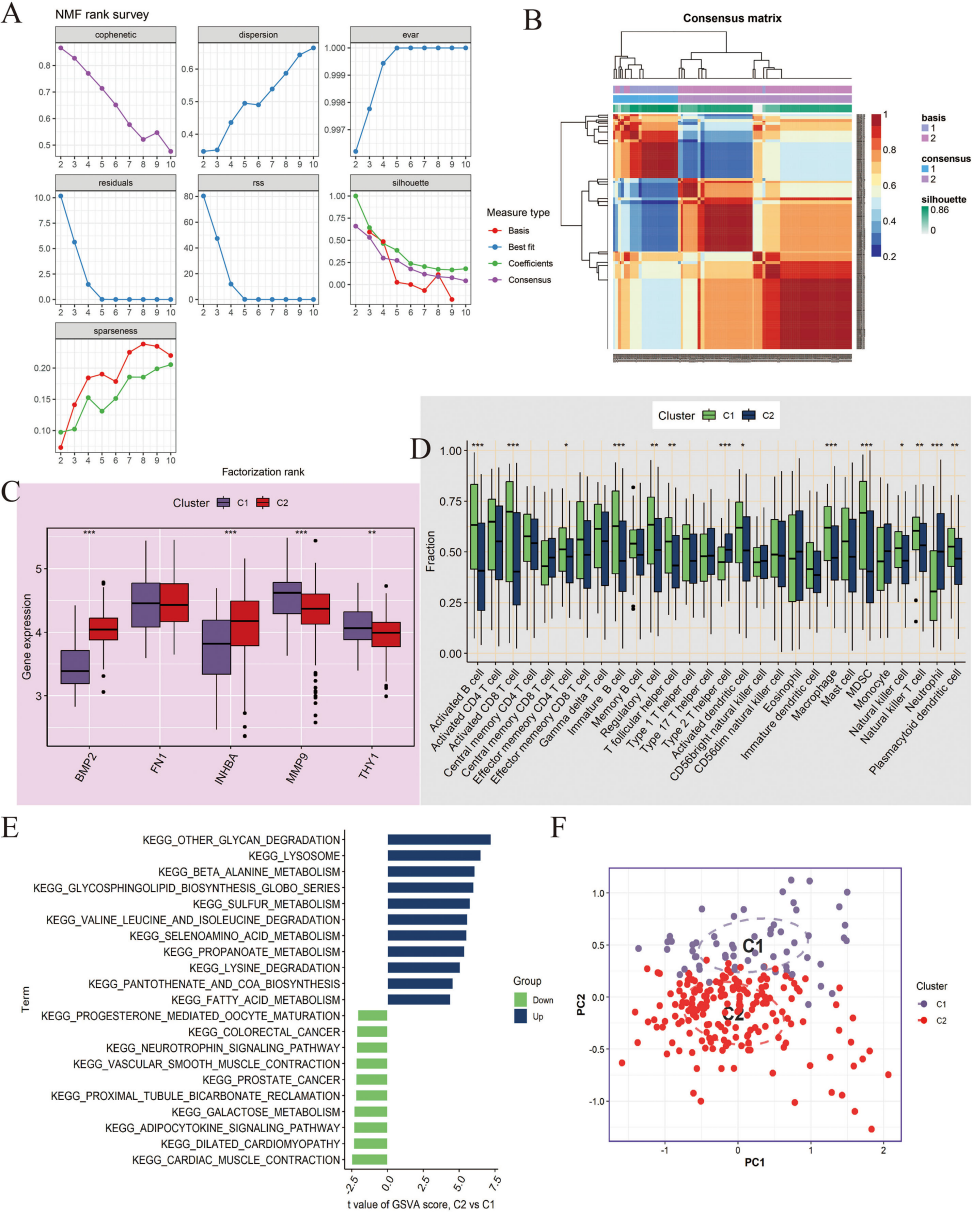
Molecular subtyping analysis based on Non-negative Matrix Factorization (NMF) clustering in NCBI data. **(A, B)** NMF clustering of tumor samples (n=260) based on 5 key genes (BMP2, FN1, INHBA, MMP9, THY1) identified two subtypes, C1 and C2. Consensus maps and cophenetic correlation coefficient were used to determine the optimal cluster number. **(C)** Boxplots showing expression differences of the key genes between C1 and C2. Differences were tested using Wilcoxon rank-sum test; *P < 0.05, **P < 0.01. **(D)** Boxplots showing the infiltration differences of 28 immune cell types between C1 and C2 subtypes, assessed by ssGSEA. Statistical significance tested by Wilcoxon rank-sum test. **(E)** GSVA enrichment analysis comparing pathway activation between C2 and C1. Enrichment scores represent normalized GSVA scores; pathways with adjusted P < 0.05 are shown. **(F)** Principal Component Analysis (PCA) demonstrating separation between C1 and C2 subtypes. ***P<0.001.

For the NCBI source data, we performed unsupervised clustering analysis using the NMF algorithm and successfully divided the patient population into two molecular subtypes (C1 and C2) with significant differences (**Figures 6A, B**). Key gene expression analysis showed that, except for FN1, the other genes had significant differences. Among them, BMP2 and INHBA had higher expression levels in C2; MMP9 and THY1 had higher expression in C1 (*P <*0.01, **Figure 6C**). Immune microenvironment analysis found that most immune cells were highly infiltrated in the C1 subtype (*P*<0.05, **Figure 6D**). GSVA enrichment analysis further revealed that C2 was significantly enriched in pro-tumor pathways such as epithelial-mesenchymal transition (EMT), angiogenesis, and inflammation (**Figure 6E**). Additionally, PCA results demonstrated that the NMF-based classification method had good discriminatory ability (**Figure 6F**).

**Figure 7:**
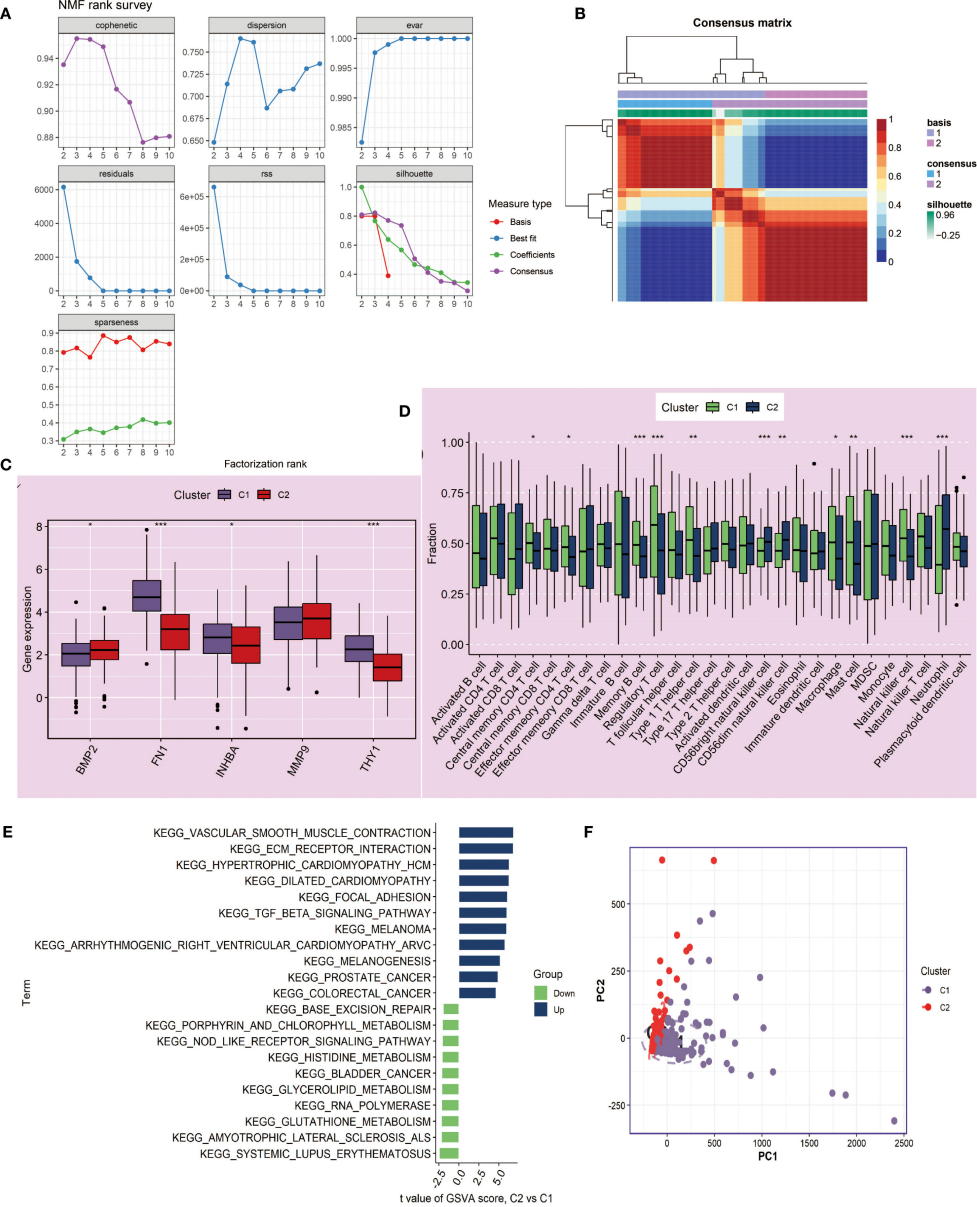
Molecular subtyping analysis based on NMF clustering in NIH data. **(A, B)** NMF clustering of tumor samples (n=241) based on 5 key genes identified two subtypes, C1 and C2. Cluster number was determined based on consensus matrices and cophenetic coefficient. **(C)** Boxplots showing expression differences of key genes between C1 and C2 subtypes. Statistical testing by Wilcoxon rank-sum test; *P < 0.05, **P < 0.01. **(D)** Boxplots showing differences in 28 immune cell infiltrations between C1 and C2, quantified by ssGSEA. Statistical significance assessed with Wilcoxon rank-sum test. **(E)** GSVA enrichment analysis comparing C2 versus C1 subtypes. Pathways with adjusted P < 0.05 are displayed; enrichment scores correspond to normalized GSVA scores. **(F)** PCA confirming discriminative ability of the NMF-based classification. ***P<0.001.

At the same time, we performed clustering analysis on the NIH source data and successfully obtained C1 and C2 subtypes (**Figures 7A, B**). We also compared the differences in key gene expression and immune cell infiltration between the two subtypes. The results showed that MMP9 had no significant difference between C1 and C2, and the other genes, except for BMP2, all had significant differences, with higher expression in C1 (*P*<0.05, **Figure 7C**). Box plot observations revealed that CD56bright natural killer cells, CD56dim natural killer cells, and Neutrophils had higher expression levels in C2, while other statistically significant immune cells were more likely to be highly expressed in C1 (*P*<0.05, **Figure 7D**). Furthermore, C2 showed significant upregulation in cancer-related pathways and downregulation in metabolism and immune-related pathways compared to C1 (**Figure 7E**). Finally, PCA analysis again confirmed the reliability of the subtyping (**Figure 7F**).

### 3.6 Immune microenvironment features and correlation analysis of key genes with immune cells

**Figure 8:**
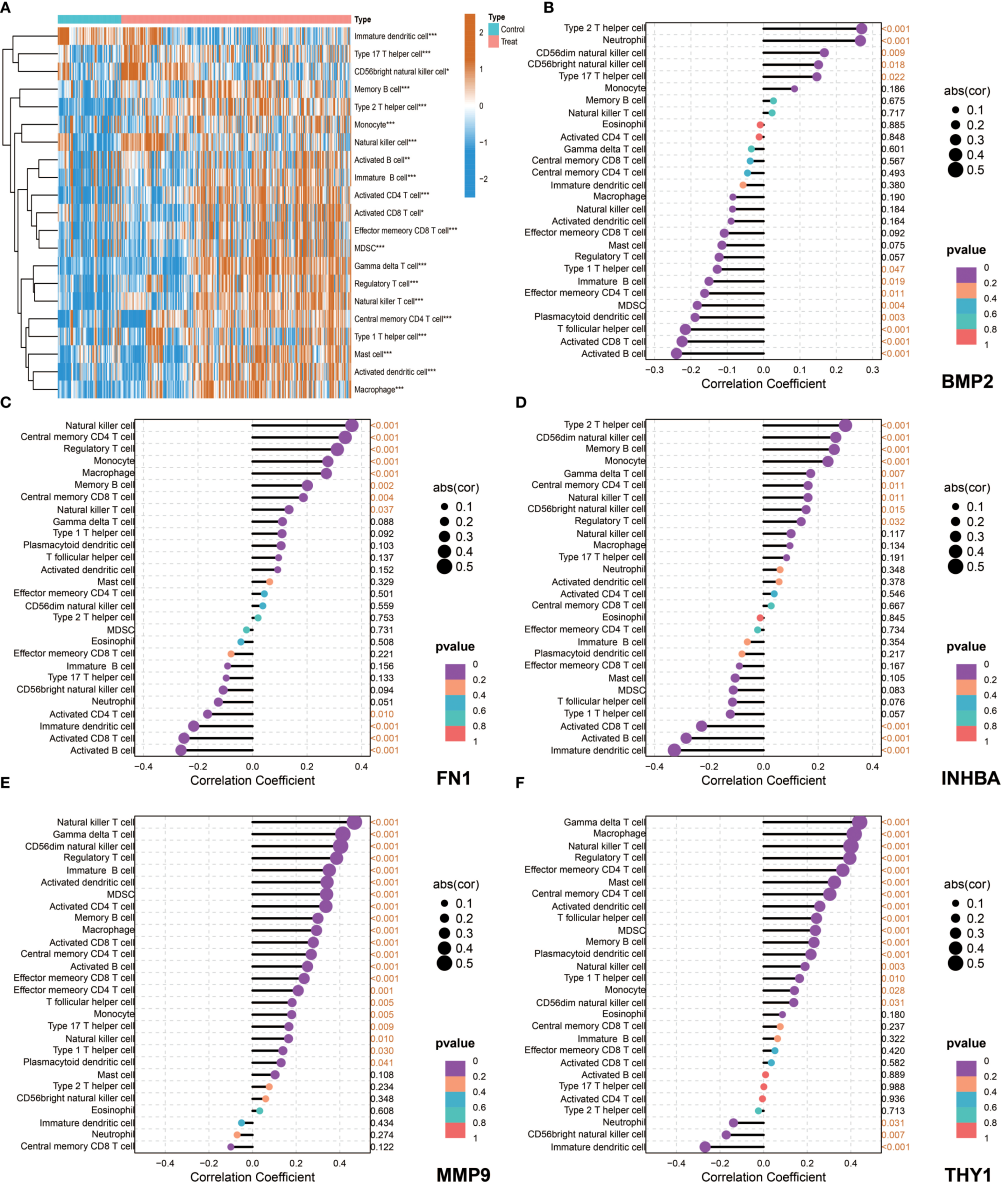
Immune microenvironment characteristics and correlation analysis between key genes and immune cells in NCBI data. **(A)** single-sample Gene Set Enrichment Analysis (ssGSEA), heatmap showing the difference in immune cell infiltration between the Control group and the Tumor group. **(B–F)** Correlation analysis between the five key genes (BMP2, FN1, INHBA, MMP9, and THY1) and immune cells in tumor patients.

**Figure 9:**
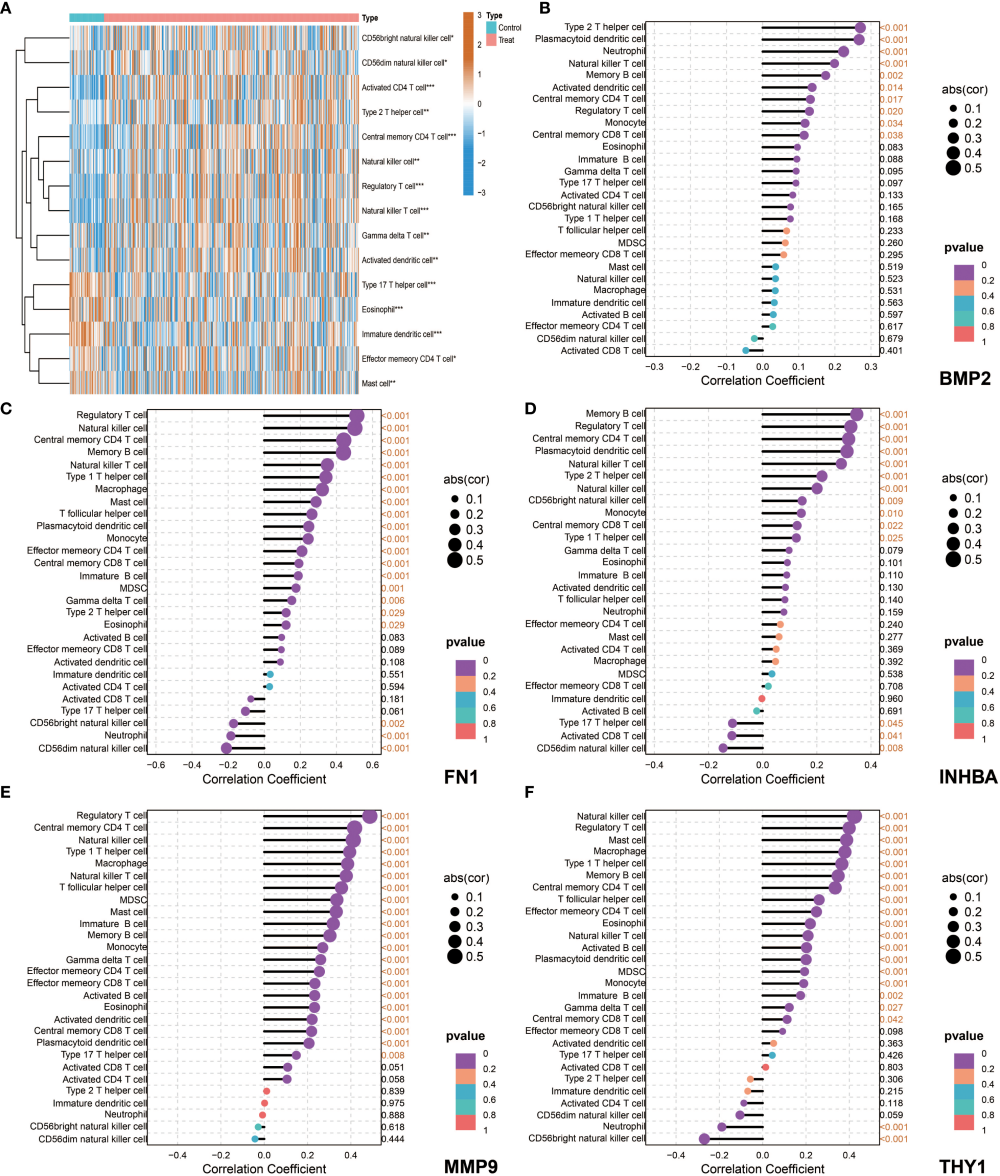
Immune microenvironment characteristics and correlation analysis between key genes and immune cells in NIH data. **(A)** ssGSEA, heatmap showing the difference in immune cell infiltration between the Control group and the Tumor group. **(B–F)** Correlation analysis between the five key genes (BMP2, FN1, INHBA, MMP9, and THY1) and immune cells in tumor patients.

The ssGSEA analysis results showed that most immune cells were highly infiltrated in OSCC patients in both the NCBI source data and NIH source data (**Figures 8A**, **9A**). Further correlation analysis showed that the 5 key genes (BMP2, FN1, INHBA, MMP9, and THY1) were closely related to the infiltration levels of various immune cells to varying degrees (**Figures 8B–F**, **9B–F**). In summary, BMP2 showed a significant positive correlation with Type 2 T helper cells and Neutrophils (*P*<0.001); FN1 showed a significant positive correlation with Natural killer cells and Central memory CD4 T cells (*P*<0.001); INHBA showed a significant positive correlation with Memory B cells (*P*<0.001); MMP9 and THY1 both showed a significant positive correlation with Natural killer cells and Regulatory T cells (*P*<0.001); moreover, THY1 also showed a significant negative correlation with CD56bright natural killer cells (*P*<0.001).

### 3.7 MR analysis

**Figure 10:**
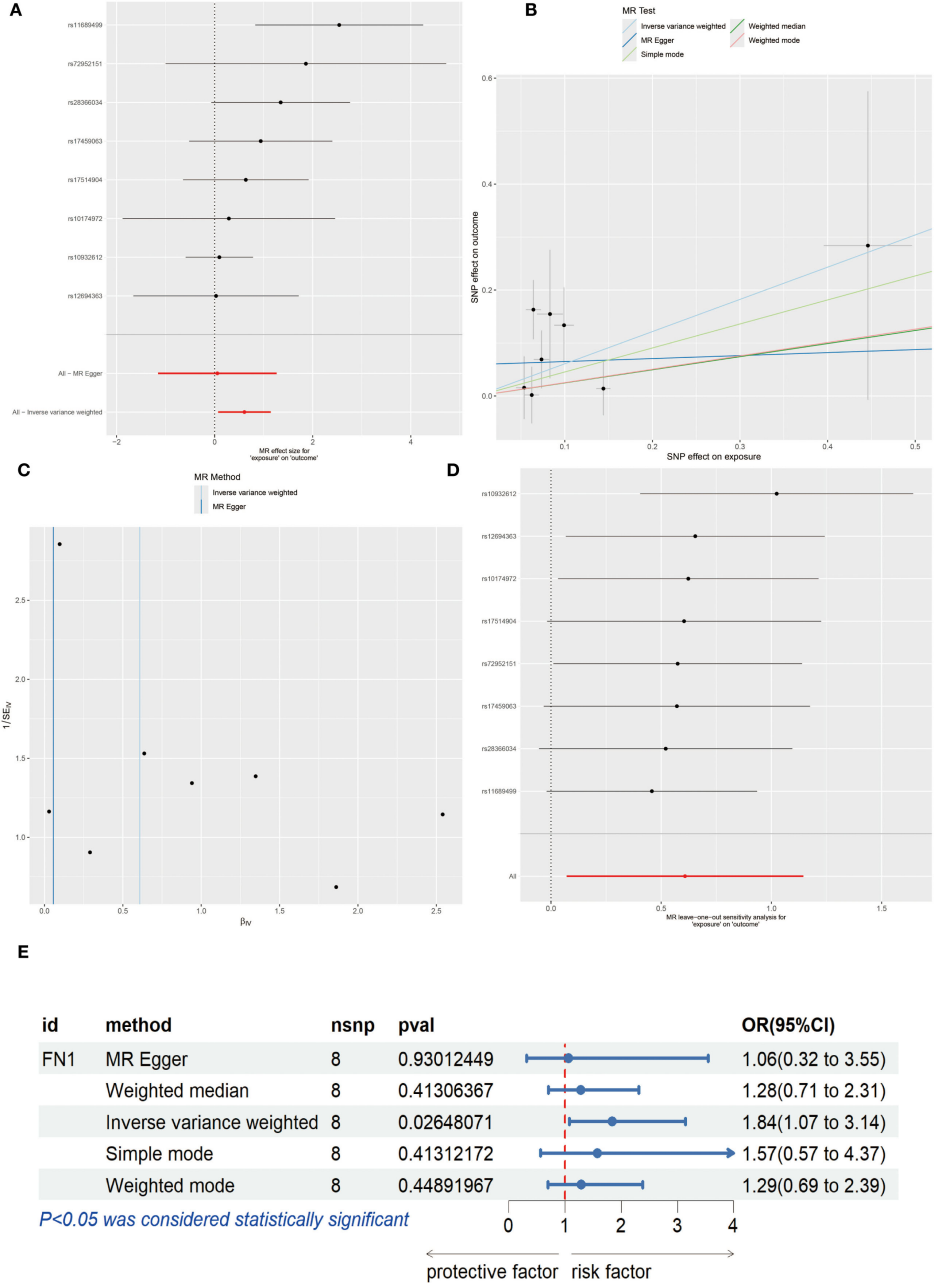
Mendelian Randomization (MR) Analysis. **(A)** Forest plot of each Single Nucleotide Polymorphism (SNP) effect on OSCC. **(B)** Scatter plot, where the x-axis represents the SNP effect on exposure and the y-axis represents the SNP effect on outcome. **(C)** Leave-one-out analysis. **(D)** Funnel plot. **(E)** Forest plot showing MR effect of FN1 on OSCC risk (OR=1.84, 95% CI 1.07–3.14, P < 0.05). Statistical significance was assessed by IVW, MR Egger, Weighted median, Weighted mode, and Simple mode methods.

To explore the causal relationship between genes and OSCC, we conducted MR analysis. First, the forest plot visually demonstrated the MR effect sizes of multiple SNPs and exposure factors on the outcome variable, with rs11689499 and rs72952151 showing significant positive associations (**Figure 10A**). The scatter plot further revealed the influence of SNPs on exposure factors and outcome variables. All five MR methods (Inverse variance weighted, MR Egger, Simple mode, Weighted median, Weighted mode) showed consistent positive linear trends, indicating a positive correlation between SNPs’ influence on exposure factors and outcome variables. Among them, the Inverse variance weighted method showed the strongest positive correlation (**Figure 10B**). Next, the leave-one-out analysis validated the robustness of the analysis and potential bias, and the results showed that the overall effect estimate was relatively stable, with rs10932612 showing a high positive correlation with OSCC outcomes (**Figures 10C, D**). Finally, for FN1, we applied various MR methods, and the results showed that the Inverse variance weighted method had statistical significance and was a risk factor with an OR of 1.84 (1.07 to 3.14) (*P*<0.05, **Figure 10E**).

### 3.8 Results of qPCR-based screening of target cell lines and functional validation of FN1

**Figure 11:**
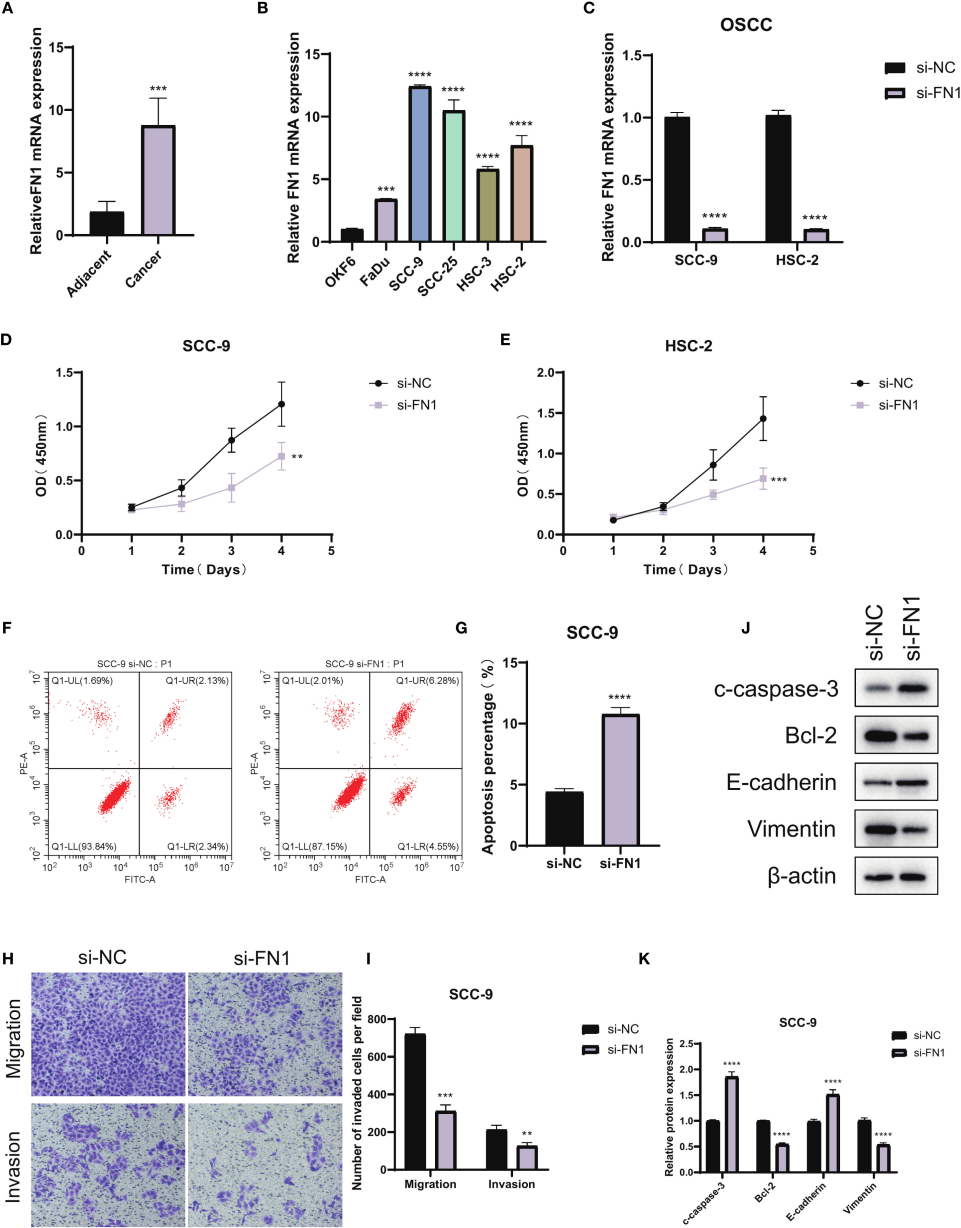
Functional validation of FN1 in OSCC. **(A)** qPCR analysis showing FN1 expression levels in OSCC tumor tissues and adjacent normal tissues. **(B)** qPCR comparison of FN1 expression in OSCC cell lines (SCC-9, HSC-2, DKF6) relative to the normal colorectal cell line FHC (n = 3 independent experiments per cell line; one-way ANOVA). **(C)** Validation of FN1 knockdown in OSCC cell lines by qRT-PCR (si-FN1 vs si-NC; n = 3, Student’s t-test; P < 0.0001). **(D, E)** CCK-8 proliferation assays showing significantly reduced cell proliferation rates in FN1-knockdown OSCC cells compared to control. **(F, G)** Flow cytometry analysis indicating a significant increase in apoptosis in FN1-depleted SCC-9 cells (n = 3, Student’s t-test; P < 0.0001). **(H, I)** Transwell assays illustrating reduced migration and invasion potential in FN1-silenced OSCC cells (n = 3, Student’s t-test; migration: P < 0.0001, invasion: P < 0.01). **(J, K)** Western blot analysis showing FN1 knockdown reduces FN1 and Bcl-2 expression, increases cleaved caspase-3 and E-cadherin, and decreases Vimentin (n = 3, Student’s t-test; P < 0.05). ***P<0.001.

The potential role of FN1, suggested as a possible prognostic marker, was further investigated through a series of functional assays in OSCC. Initially, qPCR analysis of FN1 expression in OSCC tumor tissues versus adjacent normal tissues revealed significantly elevated levels of FN1 mRNA in the tumor samples (**Figure 11A**). Subsequent qPCR profiling in OSCC cell lines showed that FN1 expression was markedly higher in SCC-9 and HSC-2 cells compared to DKF6 cells, leading to the selection of these two cell lines for further functional studies (**Figure 11B**). To explore the functional implications of FN1, it was specifically knocked down in SCC-9 and HSC-2 cells. Successful knockdown was confirmed by qRT-PCR, where FN1 mRNA levels were significantly reduced in the si-FN1 group relative to the negative control (si-NC; P < 0.0001), validating the efficacy of the knockdown (**Figure 11C**). The impact of FN1 depletion on cell proliferation was evaluated using the CCK-8 assay, which demonstrated a marked reduction in cell proliferation in FN1-silenced cells, indicating that FN1 may contribute to OSCC cell growth (**Figures 11D, E**). Flow cytometry analysis further elucidated the effect of FN1 knockdown on apoptosis. The si-FN1 group exhibited a significant increase in apoptosis rates compared to the si-NC control (P < 0.0001), confirming that FN1 depletion promotes apoptotic cell death (**Figures 11F, G**). To investigate the effects of FN1 knockdown on cellular migration and invasion, Transwell assays were performed. These assays revealed a significant reduction in both migration (P < 0.0001) and invasion (P < 0.01) in FN1-depleted cells compared to controls, suggesting that FN1 may play a role in promoting OSCC cell migration and invasion (**Figures 11H, I**). Finally, Western blot analysis was employed to assess the molecular changes following FN1 knockdown at the protein level. As expected, FN1 protein expression was significantly reduced in the si-FN1 group (P < 0.05). Additionally, the pro-apoptotic marker cleaved caspase-3 was upregulated, while the anti-apoptotic protein Bcl-2 was downregulated (P < 0.05), further supporting the notion that FN1 depletion promotes apoptosis. Furthermore, FN1 knockdown was associated with an increase in E-cadherin expression and a decrease in Vimentin expression (P < 0.05), suggesting a potential inhibitory effect on the epithelial-mesenchymal transition (EMT) process (**Figures 11J, K**).

## 4 Discussion

OSCC as one of the most common malignant tumors in the head and neck, its complex molecular mechanisms and highly heterogeneous tumor microenvironment have always been hot topics and difficult issues in research. This study systematically explored the key molecular features and immune regulatory networks of OSCC development and progression by integrating multi-omics data and machine learning algorithms. The results not only revealed the molecular heterogeneity of OSCC but also identified a series of key genes and molecular markers with potential clinical application value, providing important theoretical basis for the precise diagnosis and individualized treatment of OSCC.

In DEG analysis, a large number of differentially expressed genes closely related to OSCC were identified in both NIH and NCBI source data. Upregulated genes such as IL11, HOXA13, etc., further confirmed the important role of the inflammatory microenvironment in OSCC progression. In contrast, downregulated expression of tumor-suppressor genes such as LTF in tumor tissues may represent important molecular events in the occurrence of OSCC. Notably, the overlap of these differentially expressed genes between datasets was relatively low, which may reflect the heterogeneity of different research platforms and sample sources. As an important analysis tool in this study, WGCNA successfully constructed a gene co-expression network related to OSCC and identified several co-expression modules closely related to protein phosphorylation modifications. Among them, the MEblue and MEturquoise modules showed a high correlation with phosphorylation-related scores, indicating significant biological significance. This is because protein phosphorylation is a key regulatory mechanism in intracellular signal transduction, and its abnormal regulation is closely related to the occurrence and development of various tumors (25). The identification of these key module genes not only provides a new perspective for understanding the abnormalities in signal transduction pathways in OSCC but also lays the foundation for identifying potential therapeutic targets.

By integrating multi-omics data (differential genes from NCBI and NIH, WGCNA module genes, and phosphorylation-related genes), we successfully screened out 40 core intersecting genes. These genes play important roles in key biological processes such as cell cycle regulation, signal transduction, and metabolic reprogramming. GO analysis results showed that these genes were significantly enriched in cell division-related pathways, such as spindle formation and protein kinase complexes, which aligns with the characteristic abnormal proliferation of OSCC cells. KEGG analysis further revealed the important roles of these genes in classic tumor-related pathways such as the IL-17 signaling pathway and TGF-β signaling pathway. Notably, the significant enrichment of the p53 signaling pathway resonates with the common genomic instability features in OSCC. These findings not only deepen our understanding of the molecular mechanisms of OSCC but also provide a theoretical basis for developing new therapeutic strategies. Finally, we presented the gene expression differences of the 40 core genes in different databases.

In OSCC and related head and neck cancers, the five identified key genes demonstrate distinct but complementary roles in tumor progression. BMP2 has been shown to promote invasion and vasculogenic mimicry through CCL5 release and PI3K-AKT signaling (26, 27), highlighting its role as a driver of aggressiveness. FN1, a classic EMT marker, not only enhances proliferation, invasion, and lymph node metastasis via FAK–VEGF-C signaling but also shapes an immunosuppressive microenvironment by regulating macrophage polarization (28, 29), underscoring its dual role in tumor progression and immune modulation. INHBA, acting through the TGF-β axis, has been linked to pro-inflammatory CAFs and macrophage-driven immunosuppression (30), suggesting a central role in remodeling the tumor microenvironment. MMP9 facilitates ECM degradation and angiogenesis, partly through neutrophil-mediated MEK/ERK activation (31), reinforcing its importance in invasion and metastasis. THY1 (CD90), frequently associated with stemness and tumor recurrence in multiple cancers, may promote OSCC progression by mediating tumor–macrophage interactions and sustaining an immunosuppressive niche (32–34). Together, these findings position the five genes as critical regulators of OSCC aggressiveness and potential therapeutic targets, with direct relevance to immunotherapy and targeted intervention strategies.

Furthermore, SHAP-based interpretable machine learning analysis provided important insights into the mechanisms of key genes in the OSCC prediction model. The results showed that INHBA and MMP9 had higher SHAP values in both NIH and NCBI source data, indicating that they played a core role in model prediction, which has significant biological implications. As a subunit of activin A, INHBA mainly participates in regulating tumor occurrence and development through the TGF-β signaling pathway. Its high contribution in the model may reflect the core regulatory role of this signaling pathway in OSCC progression. The variable dependence graph further revealed the complex interactions between key genes. The study found that when multiple key genes were highly expressed simultaneously, their impact on the model prediction results was more significant. This synergistic effect may reflect the activation of multiple signaling pathways in OSCC, suggesting potential molecular mechanisms behind the tumor’s high heterogeneity. Moreover, in the analysis of accumulated contributions to model predictions, INHBA showed the most significant negative contribution in the NCBI source data, which is consistent with its function in promoting tumor invasion and metastasis in previous studies, suggesting that its high expression may predict a worse clinical outcome. BMP2 was the only gene showing a positive contribution, and its bone-forming properties may play a unique role in OSCC’s local invasion process. This positive-negative regulatory balance provides new perspectives for analyzing the molecular regulatory network of OSCC. In NIH source data, except for BMP2, all five key genes showed a negative contribution, which may be related to the clinical characteristics of NIH source samples. Considering that the NIH source data is mainly derived from advanced cancer patients, this consistent negative contribution may reflect the synergistic pro-cancer effect of these genes in the later stages of tumor progression. Model performance evaluation results showed that the RF algorithm performed best in multiple evaluation metrics such as AUC, Recall, Precision, and F1_Score. This result may stem from RF’s good adaptability to high-dimensional genomic data and its ability to capture feature interactions. It is worth noting that the validation results of RF in NIH source data were highly consistent with those in NCBI source data, which not only further validated the robustness of the model but also significantly enhanced the credibility of the research conclusions. The multi-dataset cross-validation strategy used in this study effectively reduced the bias risk from a single data source and provided more reliable and compelling evidence support for key gene screening.

Molecular Subtyping Analysis is another important component of this study. Using the NMF clustering algorithm, we successfully classified OSCC patients into two molecular subtypes, C1 and C2, which showed significant differences. These two subtypes exhibited distinct differences in key gene expression patterns, tumor immune microenvironment composition, and functional pathway enrichment. Specifically, C1 displayed a significant “immune-hot” feature, characterized by high infiltration of various immune cell subsets, such as regulatory T cells and NK cells, suggesting that patients in this subtype may be more sensitive to immune checkpoint inhibitor treatments and have potential clinical therapeutic value. In contrast, subtype C2 was marked by significantly enhanced EMT features and activation of angiogenesis-related pathways, which are closely associated with tumor invasiveness and poor prognosis. This molecular subtyping not only reveals the high heterogeneity of OSCC at the molecular level but also provides an important basis for the development of personalized treatment strategies. Finally, PCA analysis further validated the rationality of the molecular subtyping, offering crucial insights into the molecular mechanisms of each subtype.

Subsequently, tumor microenvironment analysis revealed the complexity of the OSCC immune landscape. Using the ssGSEA method, we systematically evaluated the infiltration of various immune cell types in OSCC and found significant correlations between key genes and specific immune cell subsets. For example, the positive correlation between BMP2 and neutrophils may reflect its role in inflammation microenvironment formation; the negative correlation between THY1 and NK cells may represent an immune evasion mechanism. These findings not only enrich our understanding of the OSCC immune microenvironment but also provide potential targets for the development of new immunotherapy strategies. Particularly, the positive correlation between FN1 and memory B cells suggests that tumor-associated stroma may affect tumor progression by modulating humoral immune responses, a finding worth further investigation.

Finally, MR analysis provided genetic evidence for this study. Through a systematic analysis of the association between multiple SNPs and OSCC risk, we found that rs11689499 and rs72952151 showed significant positive associations, suggesting they may be potential molecular markers of OSCC genetic susceptibility. Notably, a consistent positive association trend was observed across five different MR analysis methods (Inverse Variance Weighted, MR Egger, Simple Mode, Weighted Median, and Weighted Mode), with the strongest correlation found using Inverse Variance Weighted. The consistency of results from multiple methods significantly enhances the robustness and credibility of the research conclusions. From a biological mechanism perspective, these SNP loci may regulate the expression or functional status of the FN1 gene, affecting the extracellular matrix (ECM) remodeling process, thus promoting the occurrence and development of OSCC. Sensitivity analysis using the leave-one-out method further validated the stability of MR results, with rs10932612 consistently showing a stable positive correlation with OSCC risk, suggesting that this locus may be a key genetic variation influencing OSCC development. Importantly, the specialized MR analysis of the FN1 gene showed an odds ratio (OR) of 1.84 (95% CI: 1.07–3.14) for its role as a risk factor, a value that holds significant clinical importance in genetic epidemiology studies. FN1, which encodes fibronectin, is an essential component of the extracellular matrix, involved in various critical biological processes such as cell adhesion, migration, and signal transduction (35). Previous studies have shown that FN1 is abnormally overexpressed in various tumor tissues (36). Our study provides genetic evidence supporting a positive association between FN1 expression and OSCC risk, offering new theoretical and empirical support for considering it as a potential therapeutic target for OSCC.

Although this study has made certain innovations in multi-omics data integration and machine learning methods, there are still some aspects that need improvement. First, the study is primarily based on retrospective data from public databases, which requires validation in independent prospective cohorts. Secondly, the specific functional mechanisms of key genes still need to be further clarified through experimental research. Furthermore, the clinical significance of molecular subtypes needs to be assessed with long-term follow-up data. Future research could integrate single-cell sequencing technology to explore the heterogeneity of the tumor microenvironment in greater depth and validate the functions of key genes through organoid models or animal experiments. In terms of clinical translation, the multi-gene predictive model developed based on the results of this study holds the potential to provide new tools for the early diagnosis and prognostic evaluation of OSCC.

In summary, this study systematically reveals the molecular characteristics and immune regulatory networks of OSCC through multi-omics integration analysis and machine learning algorithms. The research not only identifies a series of key genes and molecular biomarkers with potential clinical application value but also establishes a reliable molecular subtyping system. These findings provide new insights into the pathogenesis of OSCC and lay the theoretical foundation for developing precision diagnosis and treatment strategies. Future research should focus on the clinical translation of these findings, aiming to improve the diagnosis and treatment of OSCC.

## 5 Limitations and future perspectives

This study provides novel insights into OSCC molecular mechanisms but has several limitations. Only FN1 was experimentally validated, leaving the roles of BMP2, INHBA, MMP9, and THY1 unexamined. Analyses relied on retrospective public datasets, and prospective validation is needed to confirm the clinical utility of identified biomarkers and subtypes. Some cohorts (e.g., GSE78060, GSE138206) had small sample sizes, which may introduce bias despite cross-dataset normalization. The MR analysis was limited by few valid SNPs, warranting cautious interpretation of causal inference. Finally, the mechanisms underlying molecular subtypes and their treatment associations remain unclear, highlighting the need for studies using single-cell sequencing, organoid models, and long-term clinical follow-up.

## 6 Conclusion

In this study, through systematic integration of gene expression characteristics and functional analysis, a group of phosphorylation-related molecular markers that play a key regulatory role in OSCC were identified, and their potential functions in the tumor microenvironment and signaling pathways were revealed. Molecular typing based on gene expression heterogeneity further indicates the differences in immune infiltration and cancer-promoting phenotypes among different subtypes. The research results not only deepened the understanding of the pathogenesis of OSCC, but also provided experimental evidence for exploring new diagnostic markers and therapeutic targets.

## Data Availability

The original contributions presented in the study are included in the article/supplementary material. Further inquiries can be directed to the corresponding authors.

## References

[1] Badwelan M Muaddi H Ahmed A Lee KT Tran SD. Oral squamous cell carcinoma and concomitant primary tumors, what do we know? A review of the literature. Curr Oncol. (2023) 30:3721–34. doi: 10.3390/curroncol30040283, PMID: 37185396 PMC10136780

[2] Sung H Ferlay J Siegel RL Laversanne M Soerjomataram I Jemal A. Global cancer statistics 2020: GLOBOCAN estimates of incidence and mortality worldwide for 36 cancers in 185 countries. CA Cancer J Clin. (2021) 71:209–49. doi: 10.3322/caac.21660, PMID: 33538338

[3] Chen Y Zhang X Li M Fu B Li H Yuan F. METTL1-mediated m7G modification of NEK1 mRNA promotes the proliferation of oral squamous cell carcinoma. Biochim Biophys Acta Mol Basis Dis. (2025) 1871:167961. doi: 10.1016/j.bbadis.2025.167961, PMID: 40562282

[4] Tan Y Wang Z Xu M Li B Huang Z Qin S. Oral squamous cell carcinomas: state of the field and emerging directions. Int J Oral Sci. (2023) 15:44. doi: 10.1038/s41368-023-00249-w, PMID: 37736748 PMC10517027

[5] Jagadeesan D Sathasivam KV Fuloria NK Balakrishnan V Khor GH Ravichandran M. Comprehensive insights into oral squamous cell carcinoma: Diagnosis, pathogenesis, and therapeutic advances. Pathol Res Pract. (2024) 261:155489. doi: 10.1016/j.prp.2024.155489, PMID: 39111016

[6] Chamoli A Gosavi AS Shirwadkar UP Wangdale KV Behera SK Kurrey NK. Overview of oral cavity squamous cell carcinoma: Risk factors, mechanisms, and diagnostics. Oral Oncol. (2021) 121:105451. doi: 10.1016/j.oraloncology.2021.105451, PMID: 34329869

[7] Moertel CG Dockerty MB Baggenstoss AH. Multiple primary Malignant neoplasms. I. Introduction and presentation of data. Cancer. (1961) 14:221–30. doi: 10.1002/1097-0142(196103/04)14:2<221::AID-CNCR2820140202>3.0.CO;2-6, PMID: 13771652

[8] Mroueh R Nevala A Haapaniemi A Pitkäniemi J Salo T Mäkitie AA. Risk of second primary cancer in oral squamous cell carcinoma. Head Neck. (2020) 42:1848–58. doi: 10.1002/hed.26107, PMID: 32057158

[9] Bouquot JE Whitaker SB. Oral leukoplakia–rationale for diagnosis and prognosis of its clinical subtypes or “phases. Quintessence Int. (1994) 25:133–40. 8183979

[10] Chung CS Liao LJ Wu CY Lo WC Hsieh CH Lee TH. Endoscopic screening for second primary tumors of the esophagus among head and neck cancer patients. Front Oncol. (2022) 12:906125. doi: 10.3389/fonc.2022.906125, PMID: 35747824 PMC9209650

[11] Garg M Beitler JJ. Controversies in management of the neck in head and neck cancer. Curr Treat Options Oncol. (2004) 5:35–40. doi: 10.1007/s11864-004-0004-8, PMID: 14697155

[12] Franzin R Netti GS Spadaccino F Porta C Gesualdo L Stallone G. The use of immune checkpoint inhibitors in oncology and the occurrence of AKI: where do we stand? Front Immunol. (2020) 11:574271. doi: 10.3389/fimmu.2020.574271, PMID: 33162990 PMC7580288

[13] Wang Q Sun Z Du L Xu C Wang Y Yang B. Melatonin sensitizes human colorectal cancer cells to γ-ray ionizing radiation *in vitro* and *in vivo* . Int J Mol Sci. (2018) 19:3974. doi: 10.3390/ijms19123974, PMID: 30544713 PMC6320774

[14] Lin X Wu X Gomaa A Chen J Wu L Xie X. Analysis of risk factors for multiple primary oral squamous cell carcinoma: a cohort study. Clin Oral Investig. (2020) 24:3147–55. doi: 10.1007/s00784-019-03189-0, PMID: 31903501

[15] Yao Q Bollinger C Gao J Xu D Thelen JJ. P(3)DB: an integrated database for plant protein phosphorylation. Front Plant Sci. (2012) 3:206. doi: 10.3389/fpls.2012.00206, PMID: 22973285 PMC3435559

[16] Choi J Saraf A Florens L Washburn MP Busino L. PTPN14 regulates Roquin2 stability by tyrosine dephosphorylation. Cell Cycle. (2018) 17:2243–55. doi: 10.1080/15384101.2018.1522912, PMID: 30209976 PMC6226225

[17] Tong Y Ying H Liu R Li L Bergholz J Xiao ZX. Pin1 inhibits PP2A-mediated Rb dephosphorylation in regulation of cell cycle and S-phase DNA damage. Cell Death Dis. (2015) 6:e1640. doi: 10.1038/cddis.2015.3, PMID: 25675300 PMC4669794

[18] Du S Yang B Wang X Li W-Y Lu X-H Zheng Z-H. Identification of potential leukocyte antigen-related protein (PTP-LAR) inhibitors through 3D QSAR pharmacophore-based virtual screening and molecular dynamics simulation. J Biomolecular Structure Dynamics. (2020) 38:4232–45. doi: 10.1080/07391102.2019.1676825, PMID: 31588870

[19] Ma Y Li W-Y Sun T Zhang L Lu X-H Yang B. Structure-based discovery of a specific SHP2 inhibitor with enhanced blood–brain barrier penetration from PubChem database. Bioorganic Chem. (2022) 121:105648. doi: 10.1016/j.bioorg.2022.105648, PMID: 35180489

[20] Kinehara Y Nagatomo I Koyama S Ito D Nojima S Kurebayashi R. Semaphorin 7A promotes EGFR-TKI resistance in EGFR mutant lung adenocarcinoma cells. JCI Insight. (2018) 3 (24):e123093. doi: 10.1172/jci.insight.123093, PMID: 30568033 PMC6338389

[21] Grzmil M Huber RM Hess D Frank S Hynx D Moncayo G. MNK1 pathway activity maintains protein synthesis in rapalog-treated gliomas. J Clin Invest. (2014) 124:742–54. doi: 10.1172/JCI70198, PMID: 24401275 PMC3904612

[22] Qian L Fei Q Zhang H Qiu M Zhang B Wang Q. lncRNA HOTAIR promotes DNA repair and radioresistance of breast cancer via EZH2. DNA Cell Biol. (2020) 39:2166–73. doi: 10.1089/dna.2020.5771, PMID: 33136445

[23] Oldham MC Konopka G Iwamoto K Langfelder P Kato T Horvath S. Functional organization of the transcriptome in human brain. Nat Neurosci. (2008) 11:1271–82. doi: 10.1038/nn.2207, PMID: 18849986 PMC2756411

[24] Brunet JP Tamayo P Golub TR Mesirov JP. Metagenes and molecular pattern discovery using matrix factorization. Proc Natl Acad Sci U.S.A. (2004) 101(12):4164–9. doi: 10.1073/pnas.0308531101, PMID: 15016911 PMC384712

[25] Hou S Tan J Yang B He L Zhu Y. Effect of alkylglycerone phosphate synthase on the expression profile of circRNAs in the human thyroid cancer cell line FRO. Oncol letters. (2018) 15:7889–99. doi: 10.3892/ol.2018.8356, PMID: 29731907 PMC5920571

[26] Kim MJ Kim KM Kim J Kim KN. BMP-2 promotes oral squamous carcinoma cell invasion by inducing CCL5 release. PloS One. (2014) 9:e108170. doi: 10.1371/journal.pone.0108170, PMID: 25271422 PMC4182698

[27] Zhu Q Zhang X Lu F Miao S Zhang C Liu Z. RUNX1-BMP2 promotes vasculogenic mimicry in laryngeal squamous cell carcinoma via activation of the PI3K-AKT signaling pathway. Cell Commun Signal. (2024) 22:227. doi: 10.1186/s12964-024-01605-x, PMID: 38610001 PMC11010429

[28] Morita Y Hata K Nakanishi M Omata T Morita N Yura Y. Cellular fibronectin 1 promotes VEGF-C expression, lymphangiogenesis and lymph node metastasis associated with human oral squamous cell carcinoma. Clin Exp Metastasis. (2015) 32:739–53. doi: 10.1007/s10585-015-9741-2, PMID: 26319373

[29] Zhou WH Du WD Li YF Al-Aroomi MA Yan C Wang Y. The overexpression of fibronectin 1 promotes cancer progression and associated with M2 macrophages polarization in head and neck squamous cell carcinoma patients. Int J Gen Med. (2022) 15:5027–42. doi: 10.2147/IJGM.S364708, PMID: 35607361 PMC9123938

[30] Zhao S Zhang Y Meng X Wang Y Li Y Li H. INHBA(+) macrophages and Pro-inflammatory CAFs are associated with distinctive immunosuppressive tumor microenvironment in submucous Fibrosis-Derived oral squamous cell carcinoma. BMC Cancer. (2025) 25:857. doi: 10.1186/s12885-025-14261-2, PMID: 40355814 PMC12067746

[31] Gao F Feng Y Hu X Zhang X Li T Wang Y. Neutrophils regulate tumor angiogenesis in oral squamous cell carcinoma and the role of Chemerin. Int Immunopharmacol. (2023) 121:110540. doi: 10.1016/j.intimp.2023.110540, PMID: 37354780

[32] Mancarella S Serino G Gigante I Cigliano A Ribback S Sanese P. CD90 is regulated by notch1 and hallmarks a more aggressive intrahepatic cholangiocarcinoma phenotype. J Exp Clin Cancer Res. (2022) 41:65. doi: 10.1186/s13046-022-02283-8, PMID: 35172861 PMC8851853

[33] Zhou Y Meng X He W Li X Zhao R Dong C. USF1/CD90 signaling in maintaining glioblastoma stem cells and tumor-associated macrophages adhesion. Neuro Oncol. (2022) 24:1482–93. doi: 10.1093/neuonc/noac063, PMID: 35287174 PMC10167418

[34] Al-Holou WN Wang H Ravikumar V Shankar S Oneka M Fehmi Z. Subclonal evolution and expansion of spatially distinct THY1-positive cells is associated with recurrence in glioblastoma. Neoplasia. (2023) 36:100872. doi: 10.1016/j.neo.2022.100872, PMID: 36621024 PMC9841165

[35] Que Z Xi Z Qi D Dai R Li Y Liu M. Src/FN1 pathway activation drives tumor cell cluster formation and metastasis in lung cancer: A promising therapeutic target. Sci Adv. (2025) 11:eadv7377. doi: 10.1126/sciadv.adv7377, PMID: 40632865 PMC12239954

[36] Zhang X Xiahou Z Zhao F Wu Q Nie W Wang S. Integrated multi-omics analysis reveals the immunotherapeutic significance of tumor cells with high FN1 expression in ovarian cancer. Front Mol Biosci. (2025) 12:1611964. doi: 10.3389/fmolb.2025.1611964, PMID: 40612060 PMC12221904

